# Study on the positivity rate and influencing factors of anxiety in pregnant women during the first fetal magnetic resonance examination: A cross-sectional study

**DOI:** 10.1371/journal.pone.0297177

**Published:** 2024-01-22

**Authors:** Yuping Zheng, Yun Wang, Xue Liu, Li Zhang, Hui Zhang, Juan Liu, Yang Liu, Xuesheng Li, Gang Ning

**Affiliations:** 1 Department of Radiology Nursing, West China Second University Hospital, Sichuan University, Chengdu, China; 2 Key Laboratory of Birth Defects and Related Diseases of Women and Children (Sichuan University), Ministry of Education, Chengdu, Sichuan, China; 3 Department of Radiology, West China Second University Hospital, Sichuan University, Chengdu, China; Makere University College of Health Sciences, UGANDA

## Abstract

**Objective:**

This study investigated the positive rate and related influencing factors of anxiety screening in pregnant women during the first fetal magnetic resonance examination.

**Methods:**

A total of 303 pregnant women who met the criteria for magnetic resonance pregnancy examination in a Grade III maternity hospital from December 2021 to December 2022 were included by the convenience sampling method. A cross-sectional survey was conducted before the examination using the General Situation Questionnaire and Self-rating Anxiety Scale (SAS).

**Results:**

The positive rate of anxiety was 31.02% (94/303), and the average score of anxiety was 45.71±9.84. Univariate analysis results showed that age, educational level, occupation, place of residence, per capita monthly income, and number of pregnancies were related to the anxiety status of pregnant women in the fetal magnetic resonance examination (*P*<0.05). The results of logistic regression analysis showed that the factor of college degree [OR: 2.168, 95% CI: (1.119, 4.273)] in the classification of cultural level and country factor [OR: 2.162, 95% CI: (1.066, 4.385)] in the classification of place of residence had an impact on the anxiety score of pregnant women in the fetal magnetic resonance examination (*P*<0.05).

**Conclusions:**

The positive rate of anxiety screening of pregnant women before the first prenatal magnetic resonance examination is high. A low education level and living in the countryside will increase the probability of anxiety in pregnant women during magnetic resonance examination. Based on the above research results, it is suggested that medical institutions pay attention to the mental health of pregnant women, improve mental health care services, and reduce the adverse psychological problems caused by prenatal examination.

## Introduction

Fetal magnetic resonance, as a supplementary imaging method to evaluate fetal abnormalities, can not only make a clear diagnosis of ultrasonic examination results, but also provide more diagnostic content without being limited by technical factors [1, 2], which has important prenatal diagnostic value. Foreign studies have shown that approximately 56.8% of pregnant women support magnetic resonance examination, but some women still express concerns about the impact of the examination on the fetus [3]. There is a temporary lack of research on the number of pregnant women with fetal magnetic resonance examinations in China, but previous similar studies have confirmed that fetal magnetic resonance examinations will increase the anxiety level of pregnant women and cause significant psychological distress [4]. Studies have shown that 5% to 10% of patients suffer from severe panic or claustrophobia during magnetic resonance examination [5]. In the psychological investigation of patients with magnetic resonance examination, the incidence of anxiety is as high as 45.2% [6]. Anxiety, as the main psychological reaction [7], has become an important predictor of the development of psychological problems in the process of magnetic resonance examination [8]. As a special group, pregnant women often have certain psychological pressure during the examination, which leads to physiological and psychological stress responses, such as fear, increased heart rate and elevated blood pressure [9]. Combined with the long time of examination, pregnant women show dizziness and tension, which increased the possibility of anxiety. Therefore, it is of great significance to pay attention to the anxiety of pregnant women during the fetal magnetic resonance examination.

The anxiety factors of pregnant women caused by magnetic resonance examination mainly come from the examination equipment and the subjects themselves [10]. On the one hand, the magnetic resonance examination space is closed. Due to the particularity of the examination, pregnant women need to be unable to move for a long time, and the internal environment is not comfortable. In addition, the noise generated by the equipment is large, and pregnant women lack the perception and control of the external environment, and tend to feel fear or anxiety [11]. On the other hand, women are more anxious than men in magnetic resonance examinations [12]. Due to different physiological mechanisms, physical fitness and social environments, women are more likely to show emotional behavior [13]. As a special group of women, pregnant women are worried about the impact of the examination on the fetus and the disease of the fetus. They are prone to conflict with the examination, and coupled with the strangeness of space when facing a separate examination [14], the anxiety problem is more prominent, which is not conducive to the smooth conduct of the examination [15, 16].

A meta-analysis shows that the detection rate of anxiety in prenatal pregnant women is 16% [17], and anxiety may lead to adverse maternal and infant outcomes. Studies have reported that prenatal anxiety is associated with infants’ white matter microstructure, which has a serious impact on early brain development [18]. At the same time, maternal anxiety causes fetal brain functional connectivity disorders, especially higher anxiety scores, which will affect the connection between the brainstem and the sensorimotor area, resulting in subsequent neurodevelopmental defects [19]. Meta-studies have shown that maternal anxiety has adverse birth outcomes with a risk of preterm birth and low birth weight, threatening the health of infants [20]. In addition, anxiety during pregnancy can also lead to postpartum hemorrhage, fetal distress and infection, which is not conducive to the health of pregnant women and fetuses [21]. It is very important to screen the mental health status of pregnant women with prenatal fetal magnetic resonance examination in a timely manner and put forward corresponding improvement measures according to the screening results. Therefore, on the basis of previous studies, this study conducted a cross-sectional survey of pregnant women undergoing the first fetal magnetic resonance examination through questionnaires to explore the anxiety status and influencing factors, aiming to reduce the anxiety level of pregnant women during fetal magnetic resonance examination, improve the comfort of examination, and provide an effective basis for improving the mental health of pregnant women.

## Materials and methods

In this study, a general situation questionnaire and self-rating anxiety scale were used to conduct a questionnaire survey on 303 pregnant women who underwent magnetic resonance pregnancy examination and met the criteria in the radiology clinic of a tertiary maternal and child specialist hospital in Chengdu. The survey time was from December 2021 to December 2022. This study was approved by the Ethics Committee of West China Second Hospital of Sichuan University. When filling in the questionnaire, the verbal consent of the subjects was obtained before the complete questionnaire was completed and complete data were finally obtained. If the subjects did not agree, we did not send them any questionnaires. The inclusion criteria were as follows: ①Pregnant women who participated in fetal magnetic resonance examination for the first time; ②Pregnant women who needed magnetic resonance examination due to abnormal fetal ultrasound results; ③Participants who had clear consciousness, and were able to communicate effectively, understood correctly and completed the questionnaire independently; ④Voluntary participation in the study. The exclusion criteria was pregnant women with severe mental illness. According to the requirements of statistical variable analysis, the sample size is calculated to be at least 5∼10 times the number of variables [22]. There were 14 predictors in this study, and the sample size was at least 70∼140 cases. Considering that there are approximately 20% invalid samples, the final sample is initially set at 84∼168 cases.

### General situation questionnaire

The questionnaire included pregnant women’s age, gestational weeks, educational level, occupation, place of residence (it refers to the place where pregnant women live for a long time, which is divided into three categories in this article: country, town and city), living situation, per capita monthly income, number of pregnancies, number of births, number of abortions, attention to the fetus, understanding of magnetic resonance examination, risk status of magnetic resonance examination, and demand accompanied by family members.

### Self-rating Anxiety Scale (SAS)

The SAS was developed by Zung [23], with a total of 20 items and a total score of 20∼80 points. A 4-level scoring method was adopted, including 15 positive items and 5 reverse items, which were scored according to the scoring order of 1–4 and 4–1 respectively. The standard score was rounded after the sum of the scores of each item was multiplied by 1.25. A standard score ≥50 indicates anxiety.

### Survey methods

A survey team was formed in advance and carried out the division of labor. After obtaining the consent of the subjects, the questionnaire was completed in the radiology outpatient department. The investigators adopted unified and standardized guidelines to ensure that each subject accurately understood the research content.

### Quality control

The investigation team was trained and qualified in advance. After repeated pre-investigation of all the questionnaire items, the questions were discussed and unified and standardized guidance was finally formed. After completing the questionnaire, the investigators checked whether there were any omissions in the questionnaire one by one and asked the subjects again to supplement and improve the unclear or missing points. After data collection, two researchers organized and summarized the data into an EXCELL form and double checked them to ensure the accuracy of data entry.

### Statistical analyses

SPSS 22.0 statistical software was used, frequency and percentage were used for statistical description of counting data, mean ± standard deviation was used for statistical description of measurement data, and independent sample *T* test and variance analysis were used for statistical analysis. The influencing factors of anxiety were analyzed by logistic regression analysis. The test level was α = 0.05, and *P*<0.05 indicated that the difference was statistically significant.

## Results

A total of 303 pregnant women who underwent fetal magnetic resonance examination were included in this study, with an average age of 29.92±4.09 years and an average gestational week of 28.59±4.15 weeks. The remaining data are shown in Table 1.

**Table 1:** The anxiety score of the pregnant woman in the fetal magnetic resonance examination.

Variable		N(%)	$\bar{x}\;\pm \;\text{s}$	*F*/*t*	*P value*
Age	<35	257 (84.8%)	45.04±9.00	-2.823	0.005*
	≥35	46 (15.2%)	49.43±13.11		
Gestational Weeks	<28 weeks	127 (41.9%)	45.29±10.01	-0.623	0.534
	≥28 weeks	176 (58.1%)	46.01±9.73		
Educational Level	High school degree or below	60 (19.8%)	48.45±8.38	8.546	<0.001*
	college degree	100 (33.0%)	47.47±10.19		
	bachelor degree or above	143 (47.2%)	43.32±9.65		
Occupation	manual worker	81 (26.7%)	47.81±11.49	4.832	0.009*
	mental worker	157 (51.8%)	44.05±9.06		
	Unemployed worker	65 (21.5%)	47.08±8.80		
Place of Residence	country	68 (22.4%)	49.53±11.11	7.995	<0.001*
	town	89 (29.4%)	45.78±9.64		
	city	146 (48.2%)	43.88±8.83		
Living Situation	Couples live together	152 (50.2%)	45.51±9.80	1.962	0.120
	Living with parents	76 (25.1%)	44.03±9.97		
	Living with parents-in-law	66 (21.8%)	47.95±9.97		
	Other ways of living	9 (3.0%)	46.78±5.54		
Per Capita Monthly Income	<1000	31 (10.2%)	46.74±8.29	4.541	0.004*
	1000∼2999	46 (15.2%)	48.91±12.52		
	3000∼4999	102 (33.7%)	46.75±9.87		
	>5000	124 (40.9%)	43.40±8.55		
Number of Pregnancies	1	122 (40.3%)	43.72±8.70	3.465	0.017*
	2	95 (31.4%)	47.57±11.75		
	3	52 (17.2%)	45.50±8.60		
	More than 3 times	34 (11.2%)	47.94±8.44		
Number of Births	0	203 (67.0%)	45.29±9.79	0.574	0.564
	1	93 (30.7%)	46.61±9.77		
	2 times and more	7 (2.3%)	45.71±12.70		
Number of Abortions	0	176 (58.1%)	44.57±9.53	2.845	0.060
	1	83 (27.4%)	47.31±11.21		
	2 times and more	44 (14.5%)	47.23±7.64		
Attention to The Fetus	NO	2 (0.7%)	45.00±9.90	-0.102	0.919
	YES	301 (99.3%)	45.71±9.86		
Understanding of Magnetic Resonance Examination	NO	224 (73.9%)	45.86±9.60	0.462	0.644
	YES	79 (26.1%)	45.27±10.55		
Magnetic resonance examination is risky	NO	199 (65.7%)	45.42±9.93	-0.695	0.488
	YES	104 (34.3%)	46.25±9.69		
Demand Accompanied by Family Members	NO	72 (23.8%)	44.04±9.40	-1.649	0.100
	YES	231 (76.2%)	46.23±9.94		

*, *P*<0.05, indicating that the data is statistically significant, and age, educational level, occupation, place of residence, per capita monthly income and the number of pregnancies are the influencing factors of anxiety; *F*, single factor analysis of variance; *t*, independent sample *T* test.

The positive rate of anxiety screening in this study was 31.02% (94/303), and the anxiety score was (45.71±9.84) scores. The univariate analysis results are shown in Table 1. The factors with statistical significance (*P*<0.05) in the univariate analysis were used as independent variables, and the anxiety score was used as the dependent variable (0 = without anxiety, 1 = with anxiety) for logistic regression analysis. The variables entering the regression equation were age (<35 years old = 0, ≥35 years old = 1), educational level (setting dummy variables with bachelor degree or above as reference), occupation (setting dummy variables with unemployed worker as reference), place of residence (setting dummy variables with city as reference), per capita monthly income (setting dummy variables with >5000 as reference), and number of pregnancies (setting dummy variables with more than three pregnancies as reference). Detailed data are shown in Table 2.

**Table 2:** Logistic regression analysis of the influencing factors of anxiety in pregnant women undergoing fetal magnetic resonance examination.

Variable	*Coefficient*	*SE*	*P value*	*OR*	*95%CI*
Constant	-2.764	0.847	0.001	0.063	—
college degree	0.782	0.342	0.022	2.186	(1.119,4.273)
country	0.771	0.361	0.033	2.162	(1.066,4.385)

SE, standard error; OR, odd ratio; 95% CI, 95% confidence interval.

## Discussion

The main aim of this study was to explore the positive rate of anxiety in pregnant women who underwent fetal magnetic resonance examination for the first time and the influencing factors leading to anxiety. According to the literature review, there is a lack of research on anxiety in pregnant women with fetal magnetic resonance examination. Therefore, to explore the results of this study, we mainly refer to the psychological intervention study of pregnant women in the process of magnetic resonance imaging and the psychological study of pregnant women in prenatal examination.

In this study, anxiety screening was performed on 303 pregnant women who underwent fetal magnetic resonance examination. The results showed that the positive rate of anxiety was 31.02% (94/303), which was higher than that of ordinary pregnant women (28.8%) [24] and higher than that of pregnant women with spontaneous abortion (11.43%) [25]. It can be seen that pregnant women with fetal magnetic resonance examination have a higher degree of anxiety, suggesting that health care personnel should pay attention to the mental health of the population, eliminate the influencing factors of anxiety, and take corresponding measures to intervene early.

The results of this study found that the education level and place of residence of pregnant women were risk factors for anxiety in pregnant women with fetal magnetic resonance examination. The results of this study found that pregnant women with low educational levels were more likely to have anxiety than pregnant women with high educational levels. In this study, patients with a low educational level had the highest anxiety score, which was consistent with the study of Yang [26]. The reason may be that pregnant women with low education levels receive less social support and have poor understanding of the relevant knowledge of pregnancy and the health knowledge of the fetus [27]. They cannot correctly understand the relevant information of magnetic resonance examination during pregnancy or mistakenly believe that magnetic resonance examination is to increase the efficiency of the hospital from the perspective of distrust, which leads to resistance [28]. In addition, some magnetic resonance examination costs are high, and pregnant women are worried that they cannot achieve the purpose of examination while bearing the economic burden, so the incidence of anxiety is high. An unsatisfactory living environment can also cause anxiety [29]. According to the research report [27], compared with cities, country residence is not convenient to obtain information and lacks social resources, which cannot meet the needs of physical and mental health and is more likely to lead to adverse emotions. In addition, pregnant women living in rural areas know little about magnetic resonance examinations during pregnancy due to differences in medical levels, and the noisy environment in the process causes uncomfortable reactions and aggravates anxiety. Therefore, for such pregnant women, it is necessary to give individualized pregnancy guidance, strengthen the education of health care and examination-related knowledge, improve the cognitive level of pregnant women, and reduce the possibility of anxiety.

There are also some limitations in this study: ①This study used a cross-sectional survey and only one research hospital was included. In the future, the sample size and multicenter cooperation can be increased. ②The influence of other factors on the mood of pregnant women during fetal magnetic resonance examination has not been studied. In the future, factors such as family, society, prenatal high-risk pregnant women and fetal abnormality diagnosis can be included for further research.

## Conclusion

In this study, the anxiety status of pregnant women undergoing fetal magnetic resonance examination was investigated to determine the positive rate of anxiety and its influencing factors. A low education level and living in a country will increase the probability of anxiety in pregnant women during magnetic resonance examination. The results of this study suggest that medical personnel should pay attention to assessing the mental health of pregnant women during routine pregnancy care and actively carry out examination-related health education to improve their mental health.

## References

[1] Chinese Society of Pediatric Radiology, Chinese Society of Pediatric Radiology, Chinese Society of Pediatric Radiology. Chinese expert consensus on fetal MRI. Chinese Journal of Radiology. 2020; 54(12):1153–1161. doi: 10.3760/cma.j.cn112149-20200605-00779

[2] Laifer-Narin S, Budorick NE, Simpson LL, Platt LD. Fetal magnetic resonance imaging: a review. Curr Opin Obstet Gynecol. 2007; 19(2):151–156. doi: 10.1097/GCO.0b013e32809bd978 17353684

[3] Newmark RL, Zaydlin ML, Yang A, Kuchenrither K, Wisner KL, Massey SH. Obstetric patients’ perspectives on functional magnetic neuroimaging research in pregnant women. Arch Womens Ment Health. 2019; 22(1):115–118. doi: 10.1007/s00737-018-0846-x 29687161 PMC6500439

[4] Leithner K, Pörnbacher S, Assem-Hilger E, Krampl E, Ponocny-Seliger E, Prayer D. Psychological reactions in women undergoing fetal magnetic resonance imaging. Obstet Gynecol. 2008; 111(2 Pt 1):396–402. doi: 10.1097/AOG.0b013e3181610281 18238978

[5] Meléndez JC, McCrank E. Anxiety-related reactions associated with magnetic resonance imaging examinations. JAMA. 1993; 270(6):745–747. doi: 10.1001/jama.1993.03510060091039 8336378

[6] Manning Li, Xiaozhen He, Donghong Cai. Investigation and analysis of psychological status and self-efficacy of 314 patients with nuclear magnetic resonance examination. Journal of Guangdong Medical University. 2014; 32(02):251–252.

[7] Nozzolillo R, Ercolani P, Giovagnoni A, De Nigris E, Barbini N, Mariani L, et al. Psychologic reactions of patients undergoing magnetic resonance imaging. Radiol Med. 1991;81(5):601–4. 2057583

[8] MacKenzie R, Sims C, Owens RG, Dixon AK. Patients’ perceptions of magnetic resonance imaging. Clin Radiol. 1995; 50(3):137–143. doi: 10.1016/s0009-9260(05)83042-9 7889700

[9] Jie Huang, Hudie Liang, Huihua Li. The negative emotional effects on nursing intervention of fetal magnetic resonance imaging (MRI) on the first time. Journal of Medical Imaging. 2020; 30(01):116–119.

[10] Jianghong Yu, Jinghua Shi, Weiyan Tian, Xiaojiao Zhang, Hengping Pu. Research progress of MRI-induced anxiety in subjects. Modern Medical Journal. 2021; 49(11):1355–1359.

[11] Katz RC, Wilson L, Frazer N. Anxiety and its determinants in patients undergoing magnetic resonance imaging. J Behav Ther Exp Psychiatry. 1994 ;25(2):131–134. doi: 10.1016/0005-7916(94)90005-1 7983222

[12] Al Shanbari NM, Alobaidi SF, Alhasawi R, Alzahrani AS, Bin Laswad BM, Alzahrani AA. Assessment of Anxiety Associated With MRI Examination Among the General Population in the Western Region of Saudi Arabia. Cureus, 2023; 15(2):e34531. doi: 10.7759/cureus.34531 36874299 PMC9981542

[13] Wen Hu. Investigation on mental condition of patients with MRI examinations. Chinese Journal of Disease Control and Prevention. 2014; 18(10):948–950.

[14] Shuangyue Li, Xiaoyan Liu. Psychological counseling and nursing experience of pregnant women in magnetic resonance examination. Guide of China Medicine. 2017; 15(17):224.

[15] Lulu Guo. Analysis of the influence of nursing interventions on the negative emotions of pregnant women during the first magnetic resonance examination. Heilongjiang Medical Journal. 2022; 46(20):2462–2464.

[16] Ahlander BM, Engvall J, Ericsson E. Anxiety during magnetic resonance imaging of the spine in relation to scanner design and size. Radiography (Lond). 2020; 26(2):110–116. doi: 10.1016/j.radi.2019.09.003 32052788

[17] Yan Liu, Yufeng Yu, Wenlin Yi, Yuhui Wang, Jingmei Wang. The detection rate of prenatal anxiety in China from 2010 to 2019: A Meta-analysis. Journal of Clinical Nursing in Practice. 2020; 6(10):74–79.

[18] Dean DC 3rd, Planalp EM, Wooten W, Kecskemeti SR, Adluru N, Schmidt CK, et al. Association of Prenatal Maternal Depression and Anxiety Symptoms With Infant White Matter Microstructure. JAMA Pediatr. 2018; 172(10):973–981. doi: 10.1001/jamapediatrics.2018.2132 30177999 PMC6190835

[19] De Asis-Cruz J, Krishnamurthy D, Zhao L, Kapse K, Vezina G, Andescavage N, et al. Association of prenatal maternal anxiety with fetal regional brain connectivity. JAMA Netw Open. 2020; 3(12):e2022349. doi: 10.1001/jamanetworkopen.2020.22349 33284334 PMC12549102

[20] Ding XX, Wu YL, Xu SJ, Zhu RP, Jia XM, Zhang SF, et al. Maternal anxiety during pregnancy and adverse birth outcomes: a systematic review and meta-analysis of prospective cohort studies. J Affect Disord. 2014; 159:103–110. doi: 10.1016/j.jad.2014.02.027 24679397

[21] Tingting Jiang, Yao Chen, Xuewei Zhou, Xiaomei Wang. Influence of anxiety state during pregnancy on the way of delivery, delivery process and pregnancy outcome of parturients. Chinese Journal of Hospital Statistics. 2021; 28(05):409–412.

[22] Ping Ni, Jingli Chen, Na Liu. The sample size estimation in quantitative nursing research. Chinese Journal of Nursing. 2010; 45(04):378–380.

[23] William W.K. Zung M.D. A rating instrument for anxiety disorder. Psychosomatics. 1971; 12(6):371–379. doi: 10.1016/S0033-3182(71)71479-0 5172928

[24] Guoying Zeng, Yanchen Chen, Lan Lan. Analysis of mental health status and influencing factors of pregnant women. Maternal and Child Health Care of China. 2022; 37(17):3222–3226.

[25] Qingqing Su, Yuan Zeng, Jingxian Chu, Jing Chen, Liping Wu. Effects of spontaneous abortion on psychological status of re-pregnant women. Chinese Journal of Modern Nursing. 2018; 24(34):4130–4133. doi: 10.3760/cma.j.issn.1674-2907.2018.34.009

[26] Guozhi Yang, Liping Zhu, Xiujuan Xu, Xiaojun Wang, Xian Zou, Tiancheng Song, et al. Prevalence of psychological anxiety and depression in 422 pregnant women. Practical Preventive Medicine. 2020; 27(07):861–863.

[27] Yafang Dou. Survey on mental health of pregnant women in Taizhou. Chinese Preventive Medicine. 2019; 20(10):931–936. doi: 10.16506/j.1009-6639.2019.10.010

[28] Lin Shuanghong Mei Qiongfang. Analysis of the psychological states of senile pregnant women who undergone magnetic resonance imaging examination and the intervention effect of imported health education for those elderly pregnant women. Chinese Journal of Family Planning. 2019; 27(03):382–385.

[29] Ting Yang, Hao He, Caiying Mao, Changliang Ji, Shue Zeng, Yating Hou, et al. Risk factors of prenatal depression and anxiety in pregnant women. Chinese Mental Health Journal. 2015; 29(04):246–250.

