# Supplementary material for: Study on the positivity rate and influencing factors of anxiety in pregnant women during the first fetal magnetic resonance examination: A cross-sectional study
Source: PLoS One. 2024 Jan 22;19(1):e0297177. doi: 10.1371/journal.pone.0297177 (PMC10802940; doi:10.1371/journal.pone.0297177)
Supplement: Attachment — Submitted filename: Response to Reviewers.docx [file pone.0297177.s002.docx]

Dear editors and reviewers:

We are very grateful that you and the reviewers for providing us the opportunity to revise and improve the quality of our manuscript (manuscript number: PONE-D-23-23147R1; manuscript entitled “Study on the positivity rate and influencing factors of anxiety in pregnant women during the first fetal magnetic resonance examination: a cross-sectional study”.

According to your nice suggestions, we have extensively revised the manuscript point-by-point accordingly. In this revised version, changes to our manuscript were all highlighted within the document by using red-colored text. We believe that the quality of our manuscript has been substantially improved and respectfully hope that you and reviewers approve its publication in “PLOS ONE”. If any modification is inappropriate, please do not hesitate to tell us, and we will do our best to carefully modify according to the requirements.

Thank you and best regards.

Yun Wang (corresponding author):

Address: Department of Radiology Nursing, West China Second University Hospital, Sichuan University, Key Laboratory of Obstetric and Gynecologic and Pediatric Diseases and Birth Defects of Ministry of Education

Email: 532410227@qq.com.

**According to your nice modification suggestions, we reply one by one as follows:**

**Editorial Team**

The paper has greatly improved. Please attend to the minor comments from one of the reviewers. In addition, proof-read the entire paper to correct the occassional language errors.

One of the reviewers requests to attend to some minor issues to further strengthen the paper. In addition, please proof-read the entire paper to correct the occasional grammatical errors

***Response:***

We sincerely thank you for your careful reading. Based on your nice suggestions, we tried our best to modify the language expression and language errors in the manuscript and marked them in red, but these changes will not influence the content and framework of the manuscript. In addition, regarding the revision suggestions of reviewers, we have supplemented the corresponding contents by consulting literature (Page 2 Line 22-29, Page 3 Line 1-4 in the Revised Manuscript with Track Changes). We appreciate for your warm work earnestly and hope that the correction will meet with approval.

**Reviewer #1:**

Dear authors,

Thank you for revising the manuscript. The Introduction section has been improved; however, there is a lack of elaboration on the psychological effects of the fetal magnetic resonance examination procedure based on previous studies. The current content discusses the effects of chronic anxiety during pregnancy on fetal development. In my opinion, the manuscript requires further editing.

***Response:***

Thank you very much for your kind reminder. According to your nice and professional suggestions, we have supplemented the elaboration of the related psychological effects of fetal magnetic resonance examination by consulting literature, and highlighted the importance of anxiety in fetal magnetic resonance examination in order to better link up the content of the article (Page 2 Line 22-29, Page 3 Line 1-4 in the Revised Manuscript with Track Changes). If there are any other modifications we could make, we would like very much to modify them and we really appreciate your help.
